# 1-Azido-*N*′-(phenylsulfonyl)methan­imid­amide

**DOI:** 10.1107/S1600536811037718

**Published:** 2011-09-30

**Authors:** Islam Ullah Khan, Ayyaz Mahmood, Muhammad Nadeem Arshad, Sohail Anjum Shahzad

**Affiliations:** a Materials Chemistry Laboratory, Department of Chemistry, GC University, Lahore 54000, Pakistan

## Abstract

In the title compound, C_7_H_7_N_5_O_2_S, the aromatic ring is oriented at dihedral angles of 79.46 (2) and 89.17 (2)°, respectively, with respect to the amino­(azido)­methyl and the *S*(6) six-membered ring motif generated by an intra­molecular N—H⋯O inter­action [N⋯O = 2.8901 (15) Å]. Inter­molecular N—H⋯O hydrogen bonds [N⋯O = 2.9177 (15) and 2.9757 (15) Å] generate an infinite one-dimensional network along the base vector (010).

## Related literature

For the synthesis, see: Mahmood *et al.* (2011 ▶). For related structures, see: Denny *et al.* (1980 ▶); Mahmood *et al.* (2011 ▶); Müller & Bärnighausen (1970 ▶). For graph-set notations, see: Bernstein *et al.* (1995 ▶).
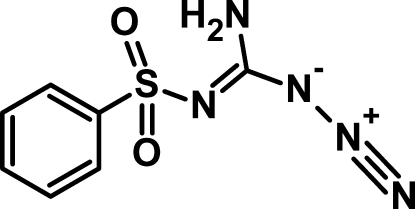


## Experimental

### 

#### Crystal data

C_7_H_7_N_5_O_2_S
*M* *_r_* = 225.24
Triclinic, 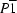

*a* = 7.0399 (2) Å
*b* = 7.1714 (2) Å
*c* = 10.3670 (3) Å
α = 90.267 (1)°
β = 98.997 (1)°
γ = 110.358 (1)°
*V* = 483.67 (2) Å^3^
*Z* = 2
Mo *K*α radiation
μ = 0.32 mm^−1^
*T* = 296 K
0.35 × 0.31 × 0.12 mm

#### Data collection

Bruker APEXII CCD diffractometer
Absorption correction: multi-scan (*SADABS*; Bruker, 2007 ▶) *T*_min_ = 0.896, *T*_max_ = 0.962
8598 measured reflections
2422 independent reflections
2246 reflections with *I* > 2σ(*I*)
*R*_int_ = 0.025

#### Refinement

*R*[*F* ^2^ > 2σ(*F* ^2^)] = 0.032
*wR*(*F* ^2^) = 0.097
*S* = 1.06
2422 reflections
143 parameters
3 restraints
H atoms treated by a mixture of independent and constrained refinement
Δρ_max_ = 0.30 e Å^−3^
Δρ_min_ = −0.30 e Å^−3^

### 

Data collection: *APEX2* (Bruker, 2007 ▶); cell refinement: *SAINT* (Bruker, 2007 ▶); data reduction: *SAINT*; program(s) used to solve structure: *SHELXS97* (Sheldrick, 2008 ▶); program(s) used to refine structure: *SHELXL97* (Sheldrick, 2008 ▶); molecular graphics: *PLATON* (Spek, 2009 ▶); software used to prepare material for publication: *WinGX* (Farrugia, 1999 ▶).

## Supplementary Material

Crystal structure: contains datablock(s) I, global. DOI: 10.1107/S1600536811037718/pv2447sup1.cif

Structure factors: contains datablock(s) I. DOI: 10.1107/S1600536811037718/pv2447Isup2.hkl

Supplementary material file. DOI: 10.1107/S1600536811037718/pv2447Isup3.cml

Additional supplementary materials:  crystallographic information; 3D view; checkCIF report

## Figures and Tables

**Table 1:** Hydrogen-bond geometry (Å, °)

*D*—H⋯*A*	*D*—H	H⋯*A*	*D*⋯*A*	*D*—H⋯*A*
N2—H1*N*⋯O1^i^	0.85 (1)	2.10 (1)	2.9177 (15)	163 (2)
N2—H2*N*⋯O2^ii^	0.84 (1)	2.23 (1)	2.9757 (15)	148 (2)
N2—H2*N*⋯O2	0.84 (1)	2.33 (2)	2.8901 (15)	125 (2)

Symmetry codes: (i) 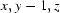; (ii) 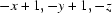.

**Fig. 1.**
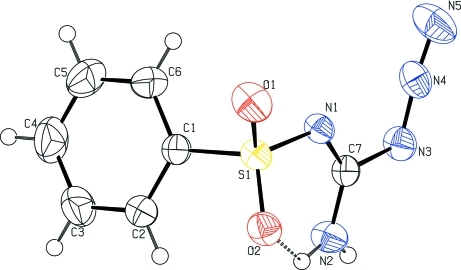
Thermal ellipsoid plot of the title compound drawn at 50% probability level showing the intramolecular hydrogen bonding via dshed line.

**Fig. 2.**
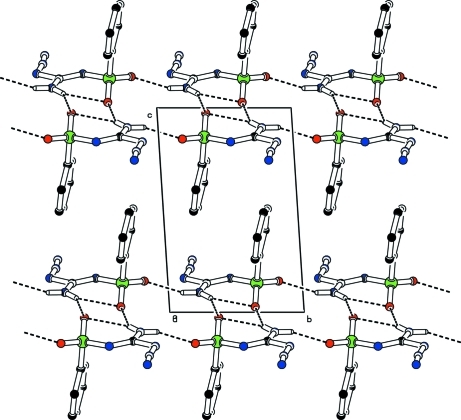
Unit cell packing diagram showing the hydrogen bonds via dshed lines, the hydrogen atoms not involved in hydrogen bonding have been omitted.

## supplementary crystallographic information

### Comment

In continuation to our studies on sulfonamide derivatives we now report the structure of the title compound which is an analogue of the compound reported earlier (Mahmood *et al.*, 2011),

In the title molecule (Fig. 1), the azido group (N3/N4/N5) carries cationic and anionic characters as observed in the previously report compound and reportedin the literature (Denny *et al.*, 1980; Müller & Bärnighausen, 1970). The bond distance N4—N5 is 1.105 (19) Å, which clearly indicates its tripple bond character (N≡N = 1.10 Å) The other bond distances, C7—N1 = 1.3105 (15) Å & C7—N3 = 1.4022 (16) Å represent C to N double and single covalent bonds, respectively. The planer amino(azido)methyl (N1/C8/N2/N3/N4/N5) moiety (r. m. s. deviation of 0.0164 A°) is oriented at a dihedral angle of 79.46 (6)° with respect to the toluene ring. The intramolecular hydrogen bond N2—H2N···O2 produces a six membered ring motif S(6) (Bernstein *et al.*, 1995) which is inclined almost perpendicular (89.17 (2)°) to the aromatic ring (C1—C6). The intermolecular hydrogen bonds are very much in accord with the corresponding hydrogen bonding reported in the previous analogue (Mahmood *et al.*, 2011) as it forms  dimers which are further connected through N—H···O type interactions and extended along the *b* axis (Table. 2, Figure. 2).

### Experimental

The titile compound was prepared in accordance with reported method (Mahmood *et al.*, 2011) and recrystalized from a mixture of methanol and ethylacetate (1:1) by slow evaporation.

### Refinement

All C—H H atoms were positioned geometrically with C_aromatic_—H = 0.93 and treated as with *U*_iso_(H) = 1.2 *U*_eq_(C). The hydrogen atoms bonded to N2 were located *via* from a fourier map and were included in the refinement with N—H distancs constrained at 0.84 (1) Å with *U*_iso_(H) = 1.5 *U*_eq_(N). The reflection 0 0 1 has been omitted in final refinement.

### Crystal data

**Table:**

C_7_H_7_N_5_O_2_S	*Z* = 2
*M**_r_* = 225.24	*F*(000) = 232
Triclinic, *P*1	*D*_x_ = 1.547 Mg m^−^^3^
Hall symbol: -P 1	Mo *K*α radiation, λ = 0.71073 Å
*a* = 7.0399 (2) Å	Cell parameters from 6153 reflections
*b* = 7.1714 (2) Å	θ = 3.0–28.5°
*c* = 10.3670 (3) Å	µ = 0.32 mm^−^^1^
α = 90.267 (1)°	*T* = 296 K
β = 98.997 (1)°	Plate, colourless
γ = 110.358 (1)°	0.35 × 0.31 × 0.12 mm
*V* = 483.67 (2) Å^3^	

### Data collection

**Table:**

Bruker APEXII CCD diffractometer	2422 independent reflections
Radiation source: fine-focus sealed tube	2246 reflections with *I* > 2σ(*I*)
graphite	*R*_int_ = 0.025
φ and ω scans	θ_max_ = 28.5°, θ_min_ = 3.0°
Absorption correction: multi-scan (*SADABS*; Bruker, 2007)	*h* = −8→9
*T*_min_ = 0.896, *T*_max_ = 0.962	*k* = −9→9
8598 measured reflections	*l* = −13→13

### Refinement

**Table:**

Refinement on *F*^2^	Secondary atom site location: difference Fourier map
Least-squares matrix: full	Hydrogen site location: inferred from neighbouring sites
*R*[*F*^2^ > 2σ(*F*^2^)] = 0.032	H atoms treated by a mixture of independent and constrained refinement
*wR*(*F*^2^) = 0.097	*w* = 1/[σ^2^(*F*_o_^2^) + (0.059*P*)^2^ + 0.1064*P*] where *P* = (*F*_o_^2^ + 2*F*_c_^2^)/3
*S* = 1.06	(Δ/σ)_max_ < 0.001
2422 reflections	Δρ_max_ = 0.30 e Å^−^^3^
143 parameters	Δρ_min_ = −0.30 e Å^−^^3^
3 restraints	Extinction correction: *SHELXL97* (Sheldrick, 2008), Fc^*^=kFc[1+0.001xFc^2^λ^3^/sin(2θ)]^-1/4^
Primary atom site location: structure-invariant direct methods	Extinction coefficient: 0.178 (13)

### Special details

**Table:**

Geometry. All s.u.'s (except the s.u. in the dihedral angle between two l.s. planes) are estimated using the full covariance matrix. The cell s.u.'s are taken into account individually in the estimation of s.u.'s in distances, angles and torsion angles; correlations between s.u.'s in cell parameters are only used when they are defined by crystal symmetry. An approximate (isotropic) treatment of cell s.u.'s is used for estimating s.u.'s involving l.s. planes.
Refinement. Refinement of *F*^2^ against ALL reflections. The weighted *R*-factor *wR* and goodness of fit *S* are based on *F*^2^, conventional *R*-factors *R* are based on *F*, with *F* set to zero for negative *F*^2^. The threshold expression of *F*^2^ > 2σ(*F*^2^) is used only for calculating *R*-factors(gt) *etc*. and is not relevant to the choice of reflections for refinement. *R*-factors based on *F*^2^ are statistically about twice as large as those based on *F*, and *R*- factors based on ALL data will be even larger.

### Fractional atomic coordinates and isotropic or equivalent isotropic displacement parameters (Å^2^)

**Table:**

	*x*	*y*	*z*	*U*_iso_*/*U*_eq_	
S1	0.29872 (4)	0.66106 (4)	0.14334 (3)	0.03096 (13)	
O1	0.22775 (17)	0.82450 (15)	0.15249 (12)	0.0501 (3)	
O2	0.37880 (15)	0.63793 (15)	0.02689 (9)	0.0407 (2)	
N4	−0.18866 (18)	0.17258 (17)	0.21333 (12)	0.0412 (3)	
N3	−0.05785 (18)	0.12367 (17)	0.16650 (12)	0.0406 (3)	
C7	0.10971 (19)	0.28677 (17)	0.14142 (11)	0.0297 (2)	
N1	0.11035 (16)	0.46635 (15)	0.16629 (10)	0.0318 (2)	
N2	0.24609 (19)	0.22835 (17)	0.09706 (13)	0.0405 (3)	
H2N	0.346 (2)	0.310 (2)	0.0691 (19)	0.061*	
H1N	0.230 (3)	0.1058 (15)	0.096 (2)	0.061*	
N5	−0.3157 (2)	0.1915 (2)	0.25610 (18)	0.0632 (4)	
C1	0.49613 (19)	0.69027 (18)	0.27813 (12)	0.0317 (3)	
C2	0.6735 (2)	0.6594 (2)	0.26000 (14)	0.0415 (3)	
H2	0.6882	0.6209	0.1775	0.050*	
C3	0.8287 (3)	0.6865 (3)	0.36578 (18)	0.0598 (5)	
H3	0.9484	0.6653	0.3549	0.072*	
C4	0.8055 (3)	0.7449 (3)	0.48716 (18)	0.0676 (5)	
H4	0.9109	0.7641	0.5579	0.081*	
C6	0.4725 (3)	0.7480 (3)	0.40091 (15)	0.0498 (4)	
H6	0.3525	0.7680	0.4125	0.060*	
C5	0.6289 (3)	0.7752 (3)	0.50550 (17)	0.0650 (5)	
H5	0.6150	0.8141	0.5882	0.078*	

### Atomic displacement parameters (Å^2^)

**Table:**

	*U*^11^	*U*^22^	*U*^33^	*U*^12^	*U*^13^	*U*^23^
S1	0.03183 (19)	0.02277 (18)	0.04005 (19)	0.01043 (12)	0.00916 (12)	0.00792 (11)
O1	0.0508 (6)	0.0279 (5)	0.0787 (8)	0.0209 (5)	0.0146 (5)	0.0126 (5)
O2	0.0400 (5)	0.0447 (6)	0.0364 (5)	0.0115 (4)	0.0113 (4)	0.0105 (4)
N4	0.0348 (6)	0.0323 (6)	0.0514 (7)	0.0040 (5)	0.0105 (5)	0.0104 (5)
N3	0.0391 (6)	0.0269 (5)	0.0526 (7)	0.0052 (5)	0.0138 (5)	0.0038 (5)
C7	0.0306 (6)	0.0250 (5)	0.0314 (5)	0.0078 (4)	0.0040 (4)	0.0044 (4)
N1	0.0294 (5)	0.0253 (5)	0.0416 (5)	0.0092 (4)	0.0095 (4)	0.0040 (4)
N2	0.0444 (7)	0.0260 (5)	0.0560 (7)	0.0136 (5)	0.0194 (5)	0.0053 (5)
N5	0.0470 (8)	0.0558 (9)	0.0908 (11)	0.0143 (7)	0.0320 (8)	0.0185 (8)
C1	0.0327 (6)	0.0232 (5)	0.0370 (6)	0.0058 (4)	0.0086 (5)	0.0025 (4)
C2	0.0364 (7)	0.0482 (8)	0.0397 (6)	0.0144 (6)	0.0074 (5)	−0.0006 (6)
C3	0.0377 (8)	0.0830 (13)	0.0552 (9)	0.0204 (8)	0.0000 (7)	0.0004 (9)
C4	0.0518 (10)	0.0894 (15)	0.0448 (8)	0.0108 (10)	−0.0068 (7)	−0.0014 (8)
C6	0.0487 (8)	0.0557 (9)	0.0453 (8)	0.0155 (7)	0.0158 (6)	−0.0042 (7)
C5	0.0696 (12)	0.0791 (13)	0.0376 (7)	0.0152 (10)	0.0099 (8)	−0.0089 (8)

### Geometric parameters (Å, °)

**Table:**

S1—O1	1.4330 (10)	C1—C2	1.3811 (19)
S1—O2	1.4419 (10)	C1—C6	1.3885 (18)
S1—N1	1.6057 (11)	C2—C3	1.382 (2)
S1—C1	1.7650 (13)	C2—H2	0.9300
N4—N5	1.1055 (19)	C3—C4	1.376 (3)
N4—N3	1.2529 (17)	C3—H3	0.9300
N3—C7	1.4022 (16)	C4—C5	1.375 (3)
C7—N1	1.3105 (15)	C4—H4	0.9300
C7—N2	1.3134 (16)	C6—C5	1.379 (3)
N2—H2N	0.837 (9)	C6—H6	0.9300
N2—H1N	0.846 (9)	C5—H5	0.9300
			
O1—S1—O2	117.23 (7)	C6—C1—S1	119.38 (11)
O1—S1—N1	105.53 (6)	C1—C2—C3	119.31 (14)
O2—S1—N1	112.47 (6)	C1—C2—H2	120.3
O1—S1—C1	107.62 (7)	C3—C2—H2	120.3
O2—S1—C1	107.32 (6)	C4—C3—C2	119.78 (16)
N1—S1—C1	106.07 (6)	C4—C3—H3	120.1
N5—N4—N3	171.37 (15)	C2—C3—H3	120.1
N4—N3—C7	113.51 (11)	C5—C4—C3	121.01 (17)
N1—C7—N2	130.53 (12)	C5—C4—H4	119.5
N1—C7—N3	118.15 (11)	C3—C4—H4	119.5
N2—C7—N3	111.31 (11)	C5—C6—C1	119.30 (15)
C7—N1—S1	121.29 (9)	C5—C6—H6	120.4
C7—N2—H2N	120.8 (13)	C1—C6—H6	120.4
C7—N2—H1N	118.7 (13)	C4—C5—C6	119.79 (16)
H2N—N2—H1N	120.5 (18)	C4—C5—H5	120.1
C2—C1—C6	120.81 (13)	C6—C5—H5	120.1
C2—C1—S1	119.80 (10)		
			
N4—N3—C7—N1	−1.19 (18)	O2—S1—C1—C6	169.50 (11)
N4—N3—C7—N2	177.67 (12)	N1—S1—C1—C6	−70.08 (12)
N2—C7—N1—S1	0.0 (2)	C6—C1—C2—C3	0.0 (2)
N3—C7—N1—S1	178.63 (9)	S1—C1—C2—C3	178.76 (13)
O1—S1—N1—C7	167.67 (11)	C1—C2—C3—C4	−0.5 (3)
O2—S1—N1—C7	38.71 (12)	C2—C3—C4—C5	0.6 (3)
C1—S1—N1—C7	−78.31 (11)	C2—C1—C6—C5	0.2 (2)
O1—S1—C1—C2	−136.24 (12)	S1—C1—C6—C5	−178.49 (14)
O2—S1—C1—C2	−9.24 (13)	C3—C4—C5—C6	−0.3 (4)
N1—S1—C1—C2	111.19 (12)	C1—C6—C5—C4	−0.1 (3)
O1—S1—C1—C6	42.49 (13)		

### Hydrogen-bond geometry (Å, °)

**Table:**

*D*—H···*A*	*D*—H	H···*A*	*D*···*A*	*D*—H···*A*
N2—H1N···O1^i^	0.85 (1)	2.10 (1)	2.9177 (15)	163.(2)
N2—H2N···O2^ii^	0.84 (1)	2.23 (1)	2.9757 (15)	148.(2)
N2—H2N···O2	0.84 (1)	2.33 (2)	2.8901 (15)	125.(2)

Symmetry codes: (i) *x*, *y*−1, *z*; (ii) −*x*+1, −*y*+1, −*z*.
